# Associations Between Age and Resting State Connectivity Are Partially Dependent Upon Cardiovascular Fitness

**DOI:** 10.3389/fnagi.2022.858405

**Published:** 2022-04-20

**Authors:** Charleen J. Gust, Erin N. Moe, Douglas R. Seals, Marie T. Banich, Jessica R. Andrews-Hanna, Kent E. Hutchison, Angela D. Bryan

**Affiliations:** ^1^Department of Psychology and Neuroscience, University of Colorado, Boulder, Boulder, CO, United States; ^2^Department of Psychiatry, University of Colorado Anschutz Medical Campus, Aurora, CO, United States; ^3^Department of Integrative Physiology, University of Colorado, Boulder, Boulder, CO, United States; ^4^Institute of Cognitive Science, University of Colorado, Boulder, Boulder, CO, United States; ^5^Department of Psychology, University of Arizona, Tucson, AZ, United States; ^6^Cognitive Science Program, University of Arizona, Tucson, AZ, United States

**Keywords:** aging, fitness, functional connectivity, frontoparietal control network, default mode network

## Abstract

Previous research suggests a marked impact of aging on structural and functional connectivity within the frontoparietal control network (FPCN) and default mode network (DMN). As aging is also associated with reductions in cardiovascular fitness, age-related network connectivity differences reported by past studies could be partially due to age-related declines in fitness. Here, we use data collected as part of a 16-week exercise intervention to explore relationships between fitness and functional connectivity. Young and older adults completed baseline assessments including cardiovascular fitness, health and functioning measures, and an fMRI session. Scan data were acquired on a Siemens 3T MRI scanner with a 32-channel head coil. Results from regression analyses indicated that average connectivity did not differ between young and older adults. However, individual ROI-to-ROI connectivity analyses indicated weaker functional correlations for older adults between specific regions in the FPCN and DMN and, critically, many of these differences were attenuated when fitness was accounted for. Taken together, findings suggest that fitness exerts regional rather than global effects on network connectivity.

## Introduction

Due to immense developments in medical technologies and treatments in recent decades, the oldest old (individuals 85+ years of age) have become the fastest growing segment of the U.S. population (National Institute on Aging and U.S. Department of Health and Human Services [DHHS], 2020). However, accompanying this enhanced longevity is an increase in the prevalence of conditions such as Alzheimer’s Disease, osteoporosis, and cardiovascular disease, among others (Jaul and Barron, 2017). Even among relatively healthy adults, the normal aging process is characterized by declines in cognitive function, especially in the domain of executive functioning (EF), and changes to emotional and motivational processing (Scheibe and Carstensen, 2010; Murman, 2015). Such changes often lead to decreased quality of life, as well as an increased susceptibility to fraud, scams, and financial distress due to impaired decision making (Denburg et al., 2007; Gamble et al., 2015).

With the utilization of functional neuroimaging techniques such as resting state functional connectivity magnetic resonance imaging (rs-fcMRI), alterations in functional coordination between brain regions, in the absence of a task, have become increasingly more apparent and robust across various populations (Zhang and Raichle, 2010). An examination of the current literature suggests at least two major resting state networks critical to executive, socioemotional, and economic functioning, namely the frontoparietal control network (FPCN) and the default mode network (DMN). The FPCN, which includes the dorsolateral prefrontal cortex (dLPFC), anterior prefrontal cortex, portions of the anterior cingulate (ACC), and dorsal precuneus, has been consistently implicated in general control processes across a wide variety of domains including socioemotional and neuroeconomic processes, as well as emotion regulation (Vincent et al., 2008; Depue et al., 2010; Ochsner et al., 2012), the selection and manipulation of information in working memory (Barnes et al., 2016), and processes related to the prioritization of information, especially under conditions of conflict (Zaki et al., 2010). Moreover, altered function of the dLPFC among clinical groups is associated with impulsive economic and risk-seeking behaviors (Meade et al., 2020).

The DMN generally comprises the medial prefrontal cortex (PFC), posterior cingulate cortex, and bilateral inferior parietal lobe, among other brain regions. It has been suggested to play an important role in introspective, self-generated processes (e.g., acting on internally generated information and evaluation of the self in reference to others) that may also influence socioemotional and neuroeconomic processes (Peters and Büchel, 2011; Andrews-Hanna, 2012). Of note, the FPCN and DMN often act in an anti-correlated manner to one another such that when individuals are engaged in cognitive control there is decreased activity in the DMN (Fox et al., 2005), although under conditions in which executive resources are needed to support internally directed cognition, these two systems may act in concert (Spreng et al., 2010).

In general, previous research suggests a marked impact of aging on structural and functional connectivity within each of these networks, but some discrepancies do exist. For example, some studies have found thickening of the ACC with age (e.g., Dotson et al., 2016) while other longitudinal analyses report significant thinning (e.g., Fjell et al., 2014). Similar discrepant results have been reported for regions of the DMN; notably, Salat et al. (2004) and Abe et al. (2008) both report age-related reductions in cortical gray matter in most anterior structures of the brain, though Salat et al. (2004) also found thickening of the medial orbitofrontal cortex with age. Findings for age-related alterations in functional connectivity are more consistent, with most studies reporting decreases in resting state connectivity within the FPCN (e.g., Geerligs et al., 2015) and DMN (e.g., Damoiseaux et al., 2008) among older adults relative to young adults (see also, review from Sala-Llonch et al., 2015). However, there are still discrepancies across studies with regard to the magnitude of age-related effects.

In a recent review paper, Andrews-Hanna et al. (2019) provide input on potential sources of variation in structural and functional connectivity findings by highlighting several challenges that often arise while conducting aging research. Broadly, they argue that previously reported age-related changes may be driven, to some degree, by motion/physiological, contextual, or motivational factors. For example, motion artifacts may partially account for discrepant results from structural studies, as older adults tend to show more variability in head motion during scanning (Savalia et al., 2017). Additionally, young and older adults may differ in their motivation for scanner-related tasks (e.g., staring at a fixation cross). Finally, though most connectivity studies exclude participants diagnosed with Alzheimer’s disease, many fail to measure the associated risk factor of Mild Cognitive Impairment (Boyle et al., 2006). Thus, age-related changes reported by previous studies may be driven in part by the presence of undetected preclinical Alzheimer’s disease in clinically normal adults.

However, it is also the case that aging is associated with reductions in physical activity (Sarkisian et al., 2005), voluntary exercise (Meisner et al., 2013), and cardiovascular fitness (Sandbakk et al., 2016). There is a growing body of research that suggests exercise in general (Li et al., 2017) and cardiovascular fitness in particular (Voss et al., 2016) have beneficial effects on brain function and connectivity. Much of this work has supported this relationship within older populations and in the domain of EF (Heyn et al., 2004). In the majority of studies comparing functional network connectivity in older vs. younger samples, the relative fitness/physical activity status of participants is not measured nor is there an explicit attempt to match participants on these variables. It is likely, however, that owing to general demographic trends the younger participants in these studies are both more active and more fit than the older participants. If true, it may be that the “age-related” network connectivity differences reported in previous studies have been overestimated and may be due—at least in part—to the effects of age-related exercise and fitness declines on connectivity rather than solely age.

The current analysis explores functional connectivity in core resting state networks strongly associated with cognition and EF in both young and older adults who were not currently meeting physical activity guidelines from the American College of Sports Medicine (American College of Sports Medicine [ACSM], 2014) at the time recruitment began for the study. The goals of this research are to replicate previous studies showing age-related differences in functional connectivity, specifically in the FPCN and DMN, and to investigate whether fitness may partially account for these differences in functional connectivity between young and older adults. Data were collected as part of a large-scale randomized controlled trial of a 16-week supervised exercise intervention designed to improve fitness, as well as cognitive, socioemotional, and economic function among sedentary adults 60 years of age and older. Given past research on age-related differences in functional and structural activity (reviewed in Sala-Llonch et al., 2015), we hypothesized that older adults would show decreased FPCN and DMN connectivity relative to young adults. Drawing from research on the neuroprotective effects of exercise (Colcombe et al., 2006), we further hypothesized that accounting for baseline fitness should result in attenuated differences in functional connectivity between young and older adults.

## Materials and Methods

The fitness and neurocognitive function data herein were collected as part of a larger intervention to increase physical activity among older adults (NCT02068612; 5R01 AG043452-05) we called Fitness, Older Adults, and Resting State Connectivity Enhancement (FORCE). Briefly, the aims of the FORCE study were to characterize associations between fitness and various domains of functioning (e.g., cognitive, emotional), as well as to examine the effects of regular exercise participation on such associations. Older adults 60 years of age and older were recruited and completed extensive assessments including cardiovascular fitness (VO_2_ peak), self-reported health and functioning measures, objective cognitive functioning measures, and a functional magnetic resonance imaging (fMRI) session. They were then randomly assigned to either low- or moderate-intensity exercise as part of a 16-week supervised exercise paradigm (results not reported here). A smaller group of young adults was recruited as a comparison for baseline fitness and neurocognitive function. These young adults completed all of the same baseline assessments but did not engage in the exercise intervention.

### Participants

Participants were recruited through community advertisements, online resources such as Craigslist, ResearchMatch, outlets commonly frequented by adults of all ages, and public records from a marketing firm. To determine eligibility, interested individuals were asked to complete a phone screen. Inclusion criteria were: (1) 25–35 years of age for young adults or 60+ years of age for older adults; (2) sedentary, defined as fewer than 80 min per week of moderate-to-vigorous intensity exercise over the past 6 months; (3) completion of the Pfeiffer Short Portable Mental Status Questionnaire (PSPMSQ; Pfeiffer, 1975) with fewer than three errors; (4) willingness to be randomly assigned (older adults only); (5) able to safely engage in moderate-intensity exercise, as assessed by a study physician; (6) completion of a VO_2_ max test without evidence of cardiac or other abnormalities; and (7) intending to remain in the Boulder-Denver area for at least 6 months (older adults only). Individuals with uncontrolled diabetes (hemoglobin A1C > 7%), uncontrolled hypertension (systolic BP ≥ 160 mmHg and/or diastolic BP ≥ 100 mmHg), bipolar disorder, schizophrenia, dementia, Alzheimer’s disease, MRI contraindications, and/or body size exceeding MRI capacity were deemed ineligible. Individuals who were pregnant or taking antipsychotic medications during screening were also excluded.

### Procedure

All procedures were reviewed and approved by the University of Colorado Boulder Institutional Review Board. Written informed consent was obtained from all participants, after which each participant completed a baseline health and function assessment, medical and MRI screening, an interview about their current fitness level, a test of physical function, and a physician supervised treadmill familiarization activity with a 12-lead EKG. All data reported herein were collected prior to older adults being randomly assigned to exercise condition.

### Magnetic Resonance Imaging Acquisition

Scan data were acquired on a Siemens 3T MRI scanner with a 32-channel head coil at the Intermountain Neuroimaging Consortium at the University of Colorado Boulder. Scan data acquired prior to April 2016 were collected on a TRIO system, while data acquired after April 2016 were collected on a Prisma Fit system. A scanner covariate was included in all analyses to control for differences in the scanner systems.

Each participant underwent a multi-echo magnetization prepared rapid acquisition with gradient echo (MPRAGE) T1 weighted anatomical scan (*TR* = 2,530 ms, *TE* = 1.64 ms, flip angle = 7°, FOV = 256 mm × 256 mm). A field map was acquired to reduce RF inhomogeneities and spatial distortion (*TR* = 400 ms, *TE* = 4.92 ms, FOV = 238 mm × 238 mm). A resting state M-EPI scan was also acquired (*TR* = 460 ms, *TE* = 29 ms, multiband acceleration factor = 8, slices = 48). During the resting state scan, participants were instructed to stare at a central fixation cross and relax for 8 min. Age-related movement artifacts were accounted for and corrected using a procedure similar to Power et al. (2012). Acquired images employed simultaneous image refocusing and multiband slice excitation (c.f., Feinberg et al., 2010). This newer method of spatial and temporal multiplexing has allowed for much faster sampling rates at < 500 ms as compared to ∼2 s while still acquiring whole brain coverage. This acquisition method also has the effect of reducing high-frequency artifacts such as physiological noise, thereby increasing the signal-to-noise ratio by 60%.

### Data Preprocessing and Analysis

Preprocessing was carried out through an SPM5-based automated analysis pipeline developed at the Mind Research Network (Bockholt et al., 2010), followed by the CONN Toolbox’s^1^ resting state functional connectivity preprocessing pipeline (Whitfield-Gabrieli and Nieto-Castanon, 2012). Preprocessing steps included removal of the first six frames to ensure intensity stabilization, skull stripping, motion realignment, segmentation of the structural image into gray matter, white matter, and cerebrospinal fluid, and identification of outlier frames, which were controlled for as first-level covariates using DVARS and framewise displacement (0.9 mm). Additional preprocessing included slice timing correction, adjustment for residual noise arising from white matter and cerebrospinal fluid, normalization of the structural image to MNI template, coregistration of the functional image to the segmented anatomical scan, and spatial smoothing (8 mm FWHM). Data were quality checked for gross artifacts or errors that may have occurred during preprocessing. Scans exhibiting excessive functional image distortion and/or magnetic field distortion were excluded (*n* = 4, all older adult participants).

Seed based rs-fcMRI analyses were completed using the CONN Toolbox. Six-millimeter diameter spherical regions of interest (ROIs) were applied to the resting state data using MNI coordinates defined by Yeo et al. (2011) (see Table 1). A band pass filter was set to 0.009–0.08 Hz to remove low and high frequency components of the signal. Spurious artifacts from the subject-specific, white matter, and CSF segmentations were regressed out, as well as signal corresponding to physiological noise. The residual time course was despiked and a bivariate correlation with no weighting was applied and used for resting state functional connectivity ROI-to-ROI analysis. A Fisher r-to-z transform was then applied to aid in normality assumptions in the higher-level analysis.

**TABLE 1:** Spherical regions of interest (ROIs) and their associated MNI coordinates.

Frontoparietal control network	
Lateral anterior prefrontal cortex	L: (–40, 50, 7); R: (40, 50, 7)
Intraparietal sulcus	L: (–43, –50, 46); R: (43, –50, 46)
Inferior temporal gyrus	L: (–57, –54, –9); R: (57, –54, –9)
Posterior-dorsal medial prefrontal cortex	L: (–5, 22, 47); R: (5, 22, 47)
Midcingulate	L: (–6, 4, 29); R: (6, 4, 29)
Posterior precuneus	L: (–4, –76, 45); R: (4, –76, 45)
Dorsolateral prefrontal cortex	L: (–45, 29, 32); R: (45, 29, 32)

**Default mode network**	

Medial prefrontal cortex	L: (–7, 49, 18); R: (7, 49, 18)
Posterior cingulate cortex	L: (–7, –52, 26); R: (7, –52, 26)
Posterior inferior parietal lobule	L: (–41, –60, 29); R: (41, –60, 29)
Superior temporal sulcus	L: (–64, –20, –9); R: (64, –20, –9)
Medial temporal lobe	L: (—25, –32, –18); R: (25, –32, –18)
Posterior-dorsal prefrontal cortex	L: (–27, 23, 48); R: (27, 23, 48)

*L, left; R, right.*

Once the analysis was completed through CONN, the first-level connectivity Fisher transformed z-scores and each pair of ROIs within both networks were extracted for each participant. The scores were averaged across all pairs of ROIs for the FPCN and DMN, separately, yielding a single mean connectivity value for each participant for each network to be used in multivariate regression analysis in R. Second-level analyses were also performed in CONN to assess the effects of age (older adults = -1, young adults = +1) on within-network, ROI-to-ROI functional connectivity in both the FPCN and DMN. The CONN toolbox uses each ROI within a network as the seed and tests the group difference in its connection to each other ROI in the network (i.e., all possible pairwise connections in a network are tested). Those results that pass correction for false discovery rate (FDR) are then reported as significantly different between the two groups. The FDR approach is utilized to control for the increased Type I error associated with a large number of tests, as it controls for a low proportion of false positives. This was followed by an additional comparison of young vs. older participants that controlled for fitness (i.e., VO_2_ peak). Both sets of results were analysis-level corrected and thresholded at p-FDR = 0.05.

### Measures

To characterize the sample, participants self-reported their age, gender, race/ethnicity, education level, and socioeconomic status at baseline.

Two measures assessed self-reported exercise behavior. Days of moderate-to-vigorous physical activity over the past 7 days was measured with the Stanford 7-Day Physical Activity Recall (PAR; Blair et al., 1985). General exercise participation was assessed using the exercise subscale of the Community Health Activities Model Program for Seniors (CHAMPS; Stewart et al., 2001). In completing the CHAMPS, participants are asked to indicate how many total hours in a typical week they participated in each of the included activities. Example activities include “Work on your car, truck, lawn mower, or other machinery” and “Jog or run.”

VO_2_ peak was assessed using an incremental graded exercise test to exhaustion with breath-by-breath gas collection (MGC Diagnostics Ultima, Saint Paul, MN) on a motorized treadmill (Full Vision Inc., Trackmaster, Newton, KS). Treadmill speed throughout the test was determined using participant heart rate (HR) and ratings of perceived exertion (RPE; Borg, 1970). While VO_2_ max is defined as “the highest rate of oxygen uptake and utilization by the body during intense, maximal exercise” (Cade et al., 2018), VO_2_ peak is the highest value of VO_2_ that an individual reaches on a specific test of high-intensity exercise. VO_2_ peak was thus used as an objective measure of fitness, as it is not uncommon for sedentary individuals to become fatigued before reaching the VO_2_ plateau requirements to measure VO_2_ max. A modified Balke protocol was used (Balke, 1963). Initial treadmill speed was selected to elicit approximately 70% of age-predicted maximum HR and an RPE rating of 13 (“somewhat hard”; Borg, 1970). Once determined, speed remained constant throughout the test and grade was increased by 2% (or 2.5% for speeds 6 mph or greater) every 2 min until exhaustion. Heart rate was continuously monitored using a 12-lead electrocardiogram (ECG). VO_2_ peak was calculated as the highest 30 s average during the test. Two participants unable to complete the VO_2_ peak assessment on the treadmill due to orthopedic limitations and/or balance concerns completed the test on a cycle ergometer (Lode Excalibur, Groningen, Netherlands). The test began at a resistance of 0 watts and increased by 20–25 watts every 2 min until exhaustion.

## Results

### Demographic Information

A total of 222 participants (42 young adults, 180 older adults) completed baseline measures of demographics, VO_2_ peak, and functional connectivity. Most participants identified as White and approximately two-thirds were female. Compared with older adults, a significantly greater proportion of young adults reported having some college and a smaller proportion had an advanced degree. Additionally, far more older adults than young adults indicated a household income of > $60,000. VO_2_ peak was significantly higher among young adults relative to older adults, as anticipated. Also as expected given our inclusion criteria, there were no significant differences in baseline exercise levels as assessed by the PAR and CHAMPS (see Table 2).

**TABLE 2:** Baseline characteristics by age group; SES, socioeconomic status; PAR, days of moderate to vigorous physical activity on the Stanford 7-Day Physical Activity Recall; CHAMPS, Community Health Activities Model Program for Seniors.

		Young adults	Older adults	Equivalence test *p*-value
Gender	% Female	57.1	65.6	*p* = 0.307
Race	% White	69.0	93.3	*p* < 0.001
	% More than one race	16.7	1.1	
	% Asian	11.9	4.4	
	% Black	2.4	0.0	
Education	% High school diploma or less	2.4	3.9	*p* = 0.069
	% Some college	28.6	13.3	
	% Bachelor’s degree	45.2	38.3	
	% Master’s degree or higher	23.8	45.0	
SES	% < $30,000	61.9	17.2	*p* < 0.001
	% $30,000–$59,999	23.8	23.9	
	% > $60,000	9.5	56.7	
Age		28.86 (3.10)	67.41 (5.57)	*p* < 0.001
VO_2_ Peak		35.96 (8.30)	24.93 (5.39)	*p* < 0.001
PAR		1.55 (1.90)	1.73 (1.95)	*p* = 0.579
CHAMPS		13.19 (6.49)	16.34 (10.05)	*p* = 0.051

*For Age, VO_2_ Peak, PAR, and CHAMPS, values are means and standard deviations are presented in parentheses.*

### Mean Within-Network Resting State Functional Connectivity Magnetic Resonance Imaging: Relationships With Age

Contrary to prior empirical studies, average FPCN connectivity did not differ between young (*M* = 0.26, *SD* = 0.08) and older adults (*M* = 0.24, *SD* = 0.07) [*t*(220) = 0.905, *p* = 0.367]. Likewise, average DMN connectivity did not differ between young (*M* = 0.35, *SD* = 0.11) and older adults (*M* = 0.34, *SD* = 0.11) [*t*(220) = 0.512, *p* = 0.610]. We next examined the association between within-network functional connectivity and continuously reported age within each group by regressing connectivity in both the FPCN and DMN on self-reported age in years.

**FIGURE 1:**
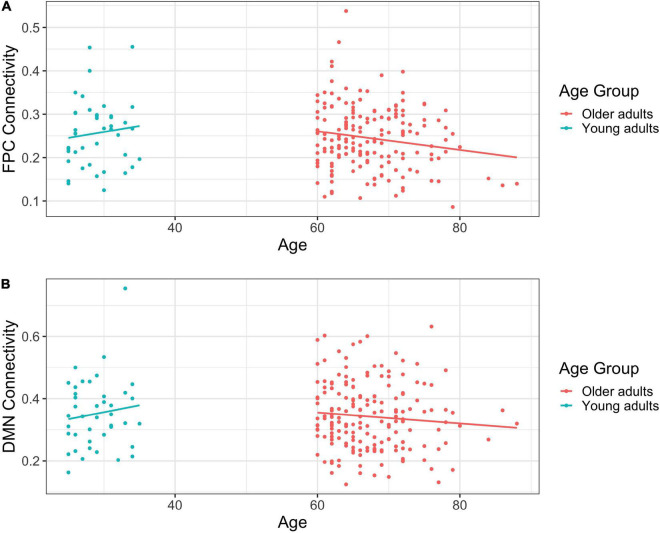
**(A)** Scatterplot of frontoparietal control network (FPCN) connectivity by self-reported age in years. **(B)** Scatterplot of default mode network (DMN) connectivity by self-reported age in years.

Among young adults, age was not significantly associated with FPCN connectivity, *b* = 0.003, *t*(40) = 0.703, η^2^ = 0.012, *p* = 0.486. In contrast, we observed a significant negative relationship between age and FPCN connectivity among older adults, *b* = –0.002, *t*(178) = –2.199, η^2^ = 0.026, *p* = 0.029, such that as age increased, connectivity decreased (see Figure 1A).

Age and DMN connectivity were unrelated among young adults, *b* = 0.004, *t*(40) = 0.794, η^2^ = 0.016, *p* = 0.432. Similarly, there was no relationship between age and DMN connectivity among older adults, *b* = –0.002, *t*(178) = –1.221, η^2^ = 0.008, *p* = 0.224 (see Figure 1B).

### Mean Within-Network Resting State Functional Connectivity Magnetic Resonance Imaging: Relationships With Fitness

To examine differences in functional connectivity by fitness, we estimated a series of models regressing FPCN and DMN connectivity on VO_2_ peak in each age group separately.

Among young adults, the association between fitness and FPCN connectivity was marginal, *b* = –0.002, *t*(40) = –1.692, η^2^ = 0.067, *p* = 0.098, such that as fitness increased, connectivity decreased. In contrast, there was no relationship between fitness and FPCN connectivity among older adults, *b* = –0.001, *t*(178) = –0.768, η^2^ = 0.003, *p* = 0.443.

Fitness and DMN connectivity were unrelated among young adults, *b* = –0.002, *t*(40) = –0.922, η^2^ = 0.021, *p* = 0.362. Likewise, fitness was not significantly associated with DMN connectivity among older adults, *b* = 0.001, *t*(178) = 0.538, η^2^ = 0.002, *p* = 0.591.

### Mean Within-Network Resting State Functional Connectivity Magnetic Resonance Imaging: Relationships With Age Controlling for Fitness

To examine differences in functional connectivity by age while controlling for fitness, we estimated a series of models regressing FPCN and DMN connectivity on age and VO_2_ peak.

For young adults, as with the initial model, age was not a significant predictor of FPCN connectivity even after controlling for fitness, *b* = 0.001, *t*(39) = 0.224, η^2^ = 0.001, *p* = 0.824. For older adults, and consistent with the initial analysis, there was still a significant relationship between age and FPCN connectivity, *b* = –0.003, *t*(177) = –2.721, η^2^ = 0.040, *p* < 0.01, after controlling for fitness. However, note that for older adults, age was negatively correlated with fitness and average FPCN connectivity, but fitness and FPCN connectivity were unrelated (see Table 3). Given the co-linearity, this result should be interpreted with caution. Fitness was also a marginal predictor of FPCN connectivity, *b* = –0.002, *t*(177) = –1.767, η^2^ = 0.017, *p* = 0.079, such that as VO_2_ peak increased, connectivity decreased.

**TABLE 3:** Correlation matrix for study variables used in regression analyses; correlations in bold are significant at the *p* < 0.05 level; FPCN, frontoparietal control network; DMN, default mode network.

	Young adults				Older adults			
Variable	1	2	3	4	1	2	3	4
1. Age	–				–			
2. VO_2_ peak	–0.27	–			–**0.38**	–		
3. Average FPCN connectivity	0.23	–0.01	–		–**0.23**	–0.03	–	
4. Average DMN connectivity	0.10	–0.03	–0.03	–	0.01	–0.03	0.12	–

As with the initial analysis, age was not a significant predictor of DMN connectivity in either age group [younger: *b* = 0.003, *t*(39) = 0.540, η^2^ = 0.007, *p* = 0.592; older: *b* = –0.002, *t*(177) = –1.095, η^2^ = 0.007, *p* = 0.275] even after controlling for fitness.

### Pairwise Region of Interest-to-Region of Interest Resting State Functional Connectivity Magnetic Resonance Imaging

To look more specifically at functional connectivity across pairs of ROIs, rather than testing differences in “overall” levels of connectivity, we further explored these effects by assessing the strength of the relationship between specific ROIs within the FPCN and DMN.

#### Pairwise Region of Interest-to-Region of Interest Resting State Functional Connectivity Magnetic Resonance Imaging: Frontoparietal Control Network

**FIGURE 2:**
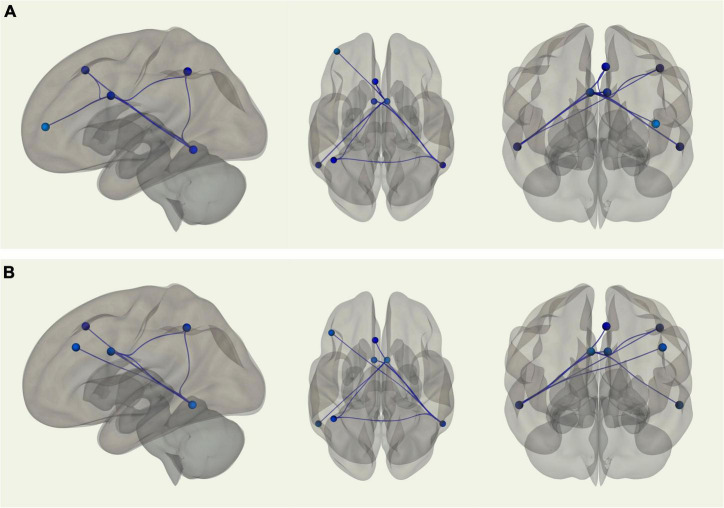
**(A)** Sagittal, transverse, and coronal images displaying functional connections between ROIs within the FPCN that were significantly different in terms of strength between young and older adults and passed correction for false discovery rate; blue connections indicate stronger functional connectivity among young adults, while red connections indicate stronger functional connectivity among older adults. Note that in this analysis there were no connections that were stronger in older adults. **(B)** Sagittal, transverse, and coronal images displaying functional connections between ROIs within the FPCN that were significantly different in terms of strength between young and older adults when controlling for VO_2_ peak and passed correction for false discovery rate; blue connections indicate stronger functional connectivity among young adults, while red connections indicate stronger functional connectivity among older adults. Note that in this analysis there were no connections that were stronger in older adults.

Using the right inferior temporal gyrus as the seed region, there was stronger connectivity between the seed and the left posterior dorsal MPFC (*p-FDR* = 0.002), left intraparietal sulcus (*p-FDR* = 0.005), left midcingulate (*p-FDR* = 0.002), and right midcingulate (*p*-FDR = 0.010) among young adults relative to older adults. Similarly, when using the left midcingulate as the seed region, young adults displayed stronger connectivity between the seed and the left inferior temporal gyrus (*p*-FDR = 0.001). When using the right midcingulate as the seed region, there was stronger connectivity among young adults between the seed and the left inferior temporal gyrus (*p*-FDR = 0.001), left intraparietal sulcus (*p*-FDR = 0.031), left posterior dorsal MPFC (*p*-FDR = 0.031), and left lateral anterior PFC (*p*-FDR = 0.031) (see Figure 2A). When controlling for VO_2_ peak, the age differences in connectivity between the left midcingulate seed region and the left inferior temporal gyrus, as well as between the right midcingulate seed region and the left posterior dorsal MPFC and left lateral anterior PFC, were no longer significant. However, correlations between the right inferior temporal gyrus seed region and the left posterior dorsal MPFC, left intraparietal sulcus, left midcingulate, and right midcingulate were still significantly different between groups, such that connectivity was still stronger in younger adults. In addition, after controlling for VO_2_ peak there was a new connection that was significantly stronger in younger adults between the right inferior temporal gyrus seed region and the left dLPFC. Correlations between the right midcingulate seed region and left inferior temporal gyrus and left intraparietal sulcus were also still significantly stronger in young adults (see Figure 2B). In sum, there were nine ROI-to-ROI connections, all involving the inferior temporal gyrus, the midcingulate, or both, that were significantly stronger in younger as opposed to older adults. After controlling for VO_2_ peak three of those connections were no longer significantly different between the groups, while one new connection favoring younger adults emerged.

#### Pairwise Region of Interest-to-Region of Interest Resting State Functional Connectivity Magnetic Resonance Imaging: Default Mode Network

**FIGURE 3:**
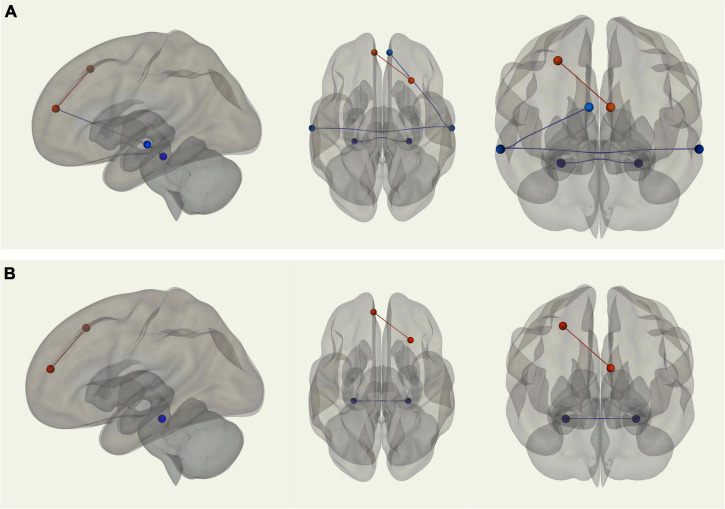
**(A)** Sagittal, transverse, and coronal images displaying functional connections between ROIs within the DMN that were significantly different in terms of strength between young and older adults and passed correction for false discovery rate; blue connections indicate stronger functional connectivity among young adults, while red connections indicate stronger functional connectivity among older adults. **(B)** Sagittal, transverse, and coronal images displaying functional connections between ROIs within the DMN that were significantly different in terms of strength between young and older adults when controlling for VO_2_ peak and passed correction for false discovery rate; blue connections indicate stronger functional connectivity among young adults, while red connections indicate stronger functional connectivity among older adults.

Using the right medial temporal lobe as the seed region, there was stronger connectivity between the seed and the left medial temporal lobe (*p-FDR* = 0.001) among young adults relative to older adults. Young adults also displayed stronger connectivity between the left and right superior temporal sulci (*p-FDR* = 0.006). Additionally, using the right superior temporal sulcus as the seed region, there was stronger connectivity between the seed and the right MPFC (*p-FDR* = 0.013) among young adults compared with older adults. In contrast, older adults displayed stronger connectivity between the left MPFC and right posterior dorsal PFC (*p-FDR* = 0.004) than young adults (see Figure 3A). When controlling for VO_2_ peak, two of these four correlations were no longer significantly different between groups. Two correlations remained significantly different; stronger connectivity for older adults between the left MPFC and right posterior dorsal PFC and stronger connectivity for younger adults between left and right medial temporal lobe (see Figure 3B).

## Discussion

In the present investigation, we sought to replicate findings from previous studies reporting age-related differences in functional connectivity and associations between fitness and connectivity, and then determine whether age-related declines in fitness might partially account for observed age differences in connectivity. In regression analyses examining associations between age, fitness, and average functional connectivity, we found somewhat perplexing results. When comparing across age groups, average connectivity in both the FPCN and DMN did not differ between young and older adults. This result is inconsistent with past research (e.g., Andrews-Hanna et al., 2007). Interestingly, in the pairwise ROI-to-ROI rs-fcMRI analyses, the strength of many of the functional correlations between specific ROIs differed between young and older adults in this sample, with older adults generally exhibiting reduced connectivity. It is therefore possible that the observed weaker functional correlations between specific regions among older adults were “washed out” when examining average connectivity across nodes in a particular network.

In contrast to the group analyses, when age was treated continuously, we observed a significant negative relationship between age and FPCN connectivity among older adults. If fitness is indeed neuroprotective (Colcombe et al., 2006), we might expect this relationship to be weaker after accounting for fitness. Instead, though fitness shared a marginal negative association with FPCN connectivity, the effect of age remained significant (and even became a bit stronger). In fairness, this is likely a statistical suppression effect given the observed correlations between age and fitness (see Table 3). More specifically, among older adults, age was significantly negatively correlated with FPCN connectivity and fitness, but FPCN connectivity and fitness themselves were uncorrelated. This is clearly problematic, given that a potential covariate should have a statistical relationship with the outcome. It also further supports our previous conclusion, namely, that using a measure of average connectivity in the context of regression analyses may not be the best method to assess the effects of fitness on age-related differences in functional connectivity.

The individual ROI-to-ROI rs-fcMRI analyses were more consistent with findings from previous studies (e.g., Andrews-Hanna et al., 2007; Damoiseaux et al., 2008), indicating weaker functional correlations for older adults between several regions in both the FPCN and DMN. Furthermore, we found that, consistent with Voss et al. (2016) controlling for fitness generally resulted in some attenuation in the differences in functional connectivity in these particular ROI-to-ROI connections between the age groups. As one example to illustrate this point, the strength of the correlation between the right MPFC and the right superior temporal sulcus no longer differed between young and older adults once fitness was accounted for. This pattern held for other regions in the DMN, as well as for several functional correlations between regions in the FPCN. This suggests that “age-related” differences in network connectivity reported by previous studies may have been overestimated because many of those studies failed to control for the effects of fitness on connectivity. Bearing in mind that fitness was unrelated to *average* FPCN and DMN connectivity in both age groups, these findings also suggest that the effects of fitness on functional connectivity do not seem to be global. In other words, fitness can account for some of the age-related differences in functional correlations between specific brain regions, but it may not be useful for explaining age-related differences in average connectivity. Future work is needed to confirm these findings and to elucidate whether there is a consistent pattern to those connections that seem to be influenced by fitness changes and those that do not. It may also be fruitful to explore this global vs. specific distinction in the extent to which other factors, such as engagement in activities that are intellectually stimulating, might explain differences in functional connectivity between young and older adults (Park et al., 2007).

Future research could also examine how connectivity *between* large-scale brain networks, in addition to alterations *within* large-scale networks, are related to both age and fitness. We primarily focused on within-network coupling in the FPCN and DMN given prior studies reporting sensitivity of those networks to age (reviewed in Sala-Llonch et al., 2015; Andrews-Hanna et al., 2019) and cardiorespiratory fitness (e.g., Voss et al., 2016), and in an attempt to reduce the number of overall statistical tests. However, older adults tend to show increased functional coupling between the FPCN and the DMN compared to young adults, as reflected in the Default-to-Executive Coupling hypothesis of aging (Turner and Spreng, 2015; Spreng and Turner, 2019). Since the FPCN is considered a brain-wide network “hub” that alters its coupling patterns with the DMN depending on one’s current goals (Spreng et al., 2010), we reasoned that between-network in the absence of an explicit task at hand (i.e., “rest”) might be especially sensitive to the co-occurring mental state of the participant, making findings difficult to interpret. Some participants may have viewed the “rest” period as an effortful externally oriented task (i.e., “remain focused on the fixation crosshair”) whereas others may have viewed the rest period as an opportunity to advance their internally oriented goals (i.e., “use this time to plan the rest of my day”). Nevertheless, a more complete analysis of relationships between fitness and brain aging, and of the relation between resting state connectivity and ongoing thought patterns, would be of broad interest in future work.

As is always the case, some limitations need to be addressed. First, it is entirely possible that those participating in the FORCE study represent an especially healthy sample of older adults, in terms of both physiology and cognitive function. Participants were recruited from the Boulder-Denver area, two cities consistently cited as being among the healthiest and most active in the United States (Warren, 2020). In addition, our inclusion criteria required participants to be engaging in fewer than 80 min of moderate-to-vigorous exercise each week, rather than being completely sedentary. Thus, the older adults in our sample may have been healthier and fitter than what is typical. A related limitation is that most adults in the current sample reported being white, educated, and of middle socioeconomic status, further restricting the generalizability of the findings. The size of the young adult sample was also relatively small. Finally, our MRI scanner was upgraded part-way through the larger study, which past studies have shown can introduce bias and therefore reduce the integrity of the results (Chen et al., 2014). Efforts were made to reduce this bias by equating critical parameters across the systems and including a covariate for scanner in our analyses.

## Conclusion

To conclude, this investigation employed a combination of analysis techniques to explore whether fitness partially accounts for important age-related differences in functional connectivity. Our initial approach involving a series of regressions using an average measure of connectivity showed that FPCN and DMN connectivity did not differ between young and older adults, and our measure of fitness was uncorrelated with average connectivity in both age groups. Seed-based rs-fcMRI analyses were more consistent with findings from previous studies (e.g., Andrews-Hanna et al., 2007), indicating weaker functional correlations between several regions in the FPCN and DMN among older adults as compared to younger adults. Critically, many of these differences were attenuated when fitness was accounted for. Taken together, our findings suggest that fitness exerts regional rather than global effects on network connectivity, which can account for some of the differences in functional connectivity across age.

## Data Availability Statement

The raw data supporting the conclusions of this article will be made available by the authors, without undue reservation.

## Ethics Statement

The studies involving human participants were reviewed and approved by the University of Colorado Boulder Institutional Review Board, Office of Research Integrity. The patients/participants provided their written informed consent to participate in this study.

## Author Contributions

DS, MB, JA-H, KH, and AB contributed to conception and design of the study. AB was responsible for funding acquisition and project administration. CG and EM performed the statistical analyses. CG, EM, and AB wrote the first draft of the manuscript. All authors contributed to manuscript revision, read, and approved the submitted version.

## Conflict of Interest

The authors declare that the research was conducted in the absence of any commercial or financial relationships that could be construed as a potential conflict of interest.

## Publisher’s Note

All claims expressed in this article are solely those of the authors and do not necessarily represent those of their affiliated organizations, or those of the publisher, the editors and the reviewers. Any product that may be evaluated in this article, or claim that may be made by its manufacturer, is not guaranteed or endorsed by the publisher.

## Footnotes

1
https://web.conn-toolbox.org/

## Supplementary Material

Supplementary Figure 1 Sagittal image displaying functional connections between ROIs within the FPCN that were significantly different in terms of strength between young and older adults and passed correction for false discovery rate; blue connections indicate stronger functional connectivity among young adults.

Supplementary Figure 2 Transverse image displaying functional connections between ROIs within the FPCN that were significantly different in terms of strength between young and older adults and passed correction for false discovery rate; blue connections indicate stronger functional connectivity among young adults.

Supplementary Figure 3 Coronal image displaying functional connections between ROIs within the FPCN that were significantly different in terms of strength between young and older adults and passed correction for false discovery rate; blue connections indicate stronger functional connectivity among young adults.

Supplementary Figure 4 Sagittal image displaying functional connections between ROIs within the FPCN that were significantly different in terms of strength between young and older adults when controlling for VO_2_ peak and passed correction for false discovery rate; blue connections indicate stronger functional connectivity among young adults.

Supplementary Figure 5 Transverse image displaying functional connections between ROIs within the FPCN that were significantly different in terms of strength between young and older adults when controlling for VO_2_ peak and passed correction for false discovery rate; blue connections indicate stronger functional connectivity among young adults.

Supplementary Figure 6 Coronal image displaying functional connections between ROIs within the FPCN that were significantly different in terms of strength between young and older adults when controlling for VO_2_ peak and passed correction for false discovery rate; blue connections indicate stronger functional connectivity among young adults.

Supplementary Figure 7 Sagittal image displaying functional connections between ROIs within the DMN that were significantly different in terms of strength between young and older adults and passed correction for false discovery rate; blue connections indicate stronger functional connectivity among young adults, while red connections indicate stronger functional connectivity among older adults.

Supplementary Figure 8 Transverse image displaying functional connections between ROIs within the DMN that were significantly different in terms of strength between young and older adults and passed correction for false discovery rate; blue connections indicate stronger functional connectivity among young adults, while red connections indicate stronger functional connectivity among older adults.

Supplementary Figure 9 Coronal image displaying functional connections between ROIs within the DMN that were significantly different in terms of strength between young and older adults and passed correction for false discovery rate; blue connections indicate stronger functional connectivity among young adults, while red connections indicate stronger functional connectivity among older adults.

Supplementary Figure 10 Sagittal image displaying functional connections between ROIs within the DMN that were significantly different in terms of strength between young and older adults when controlling for VO_2_ peak and passed correction for false discovery rate; blue connections indicate stronger functional connectivity among young adults, while red connections indicate stronger functional connectivity among older adults.

Supplementary Figure 11 Transverse image displaying functional connections between ROIs within the DMN that were significantly different in terms of strength between young and older adults when controlling for VO_2_ peak and passed correction for false discovery rate; blue connections indicate stronger functional connectivity among young adults, while red connections indicate stronger functional connectivity among older adults.

Supplementary Figure 12 Coronal image displaying functional connections between ROIs within the DMN that were significantly different in terms of strength between young and older adults when controlling for VO_2_ peak and passed correction for false discovery rate; blue connections indicate stronger functional connectivity among young adults, while red connections indicate stronger functional connectivity among older adults.
