# Supplementary figures and images for: Associations Between Age and Resting State Connectivity Are Partially Dependent Upon Cardiovascular Fitness
Source: Front Aging Neurosci. 2022 Apr 20;14:858405. doi: 10.3389/fnagi.2022.858405 (PMC9067399; doi:10.3389/fnagi.2022.858405)

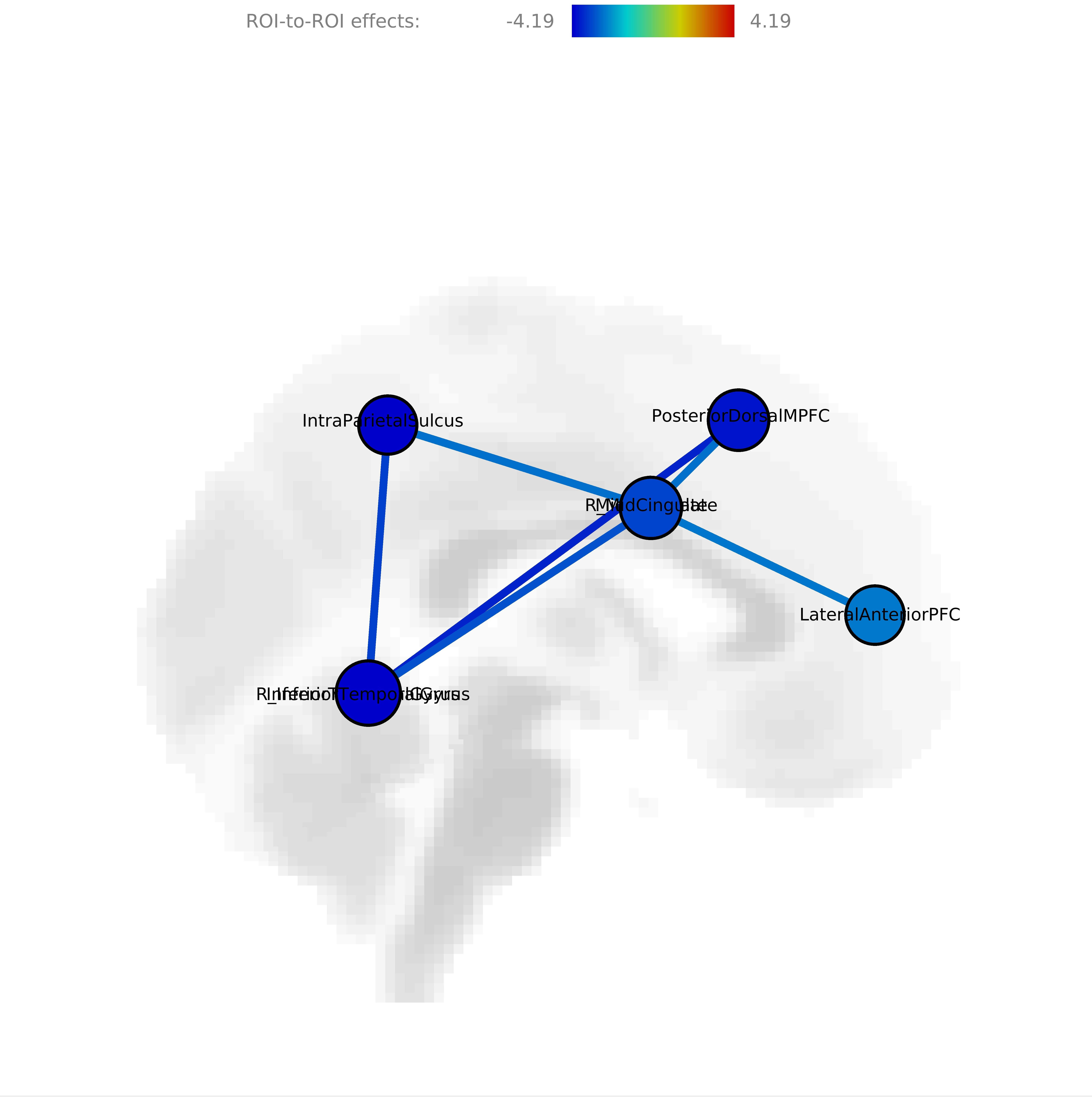

Supplement: Supplementary Figure 1 — Sagittal image displaying functional connections between ROIs within the FPCN that were significantly different in terms of strength between young and older adults and passed correction for false discovery rate; blue connections indicate stronger functional connectivity among young adults. [file Image_1.jpg]

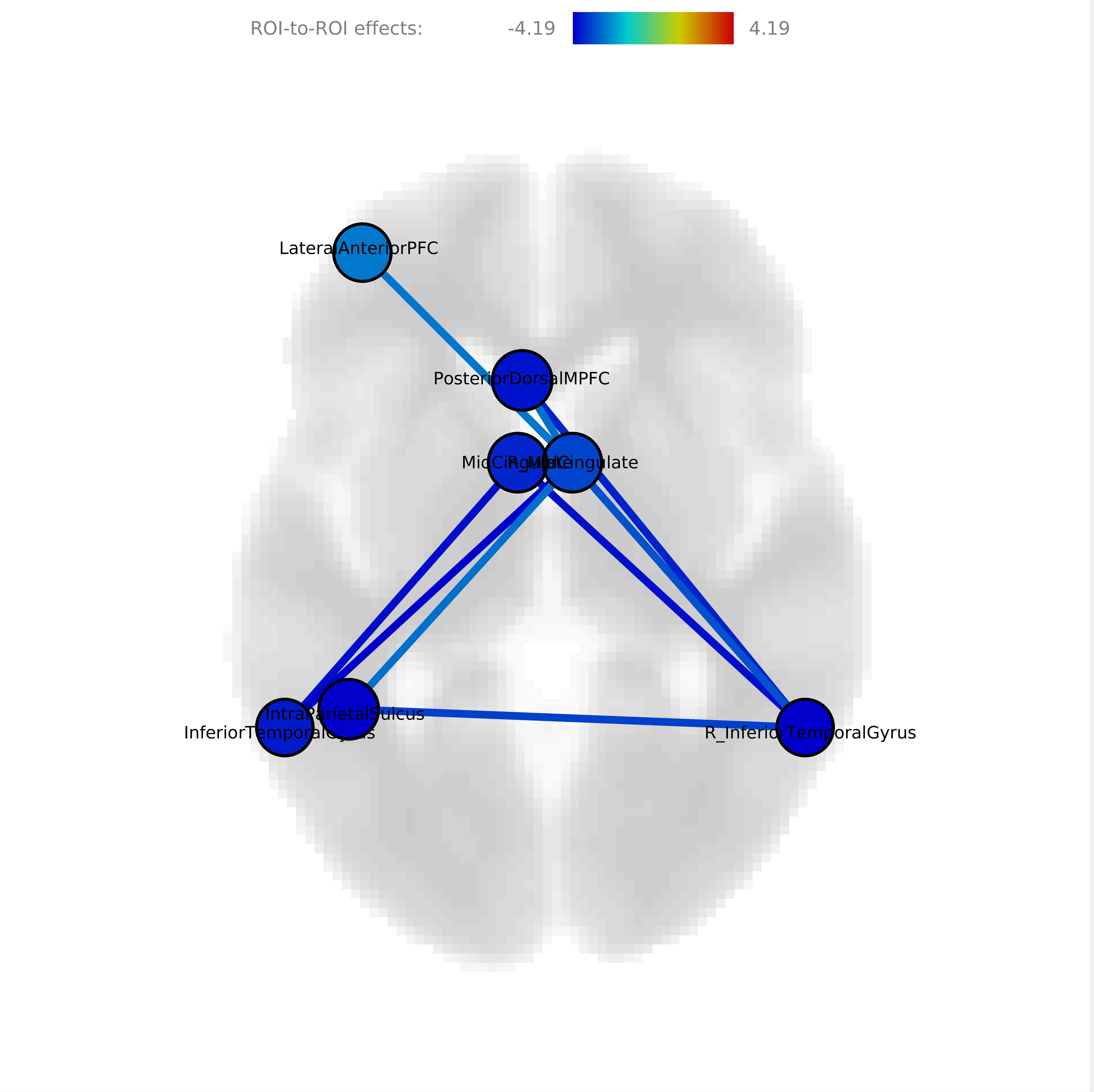

Supplement: Supplementary Figure 2 — Transverse image displaying functional connections between ROIs within the FPCN that were significantly different in terms of strength between young and older adults and passed correction for false discovery rate; blue connections indicate stronger functional connectivity among young adults. [file Image_2.jpg]

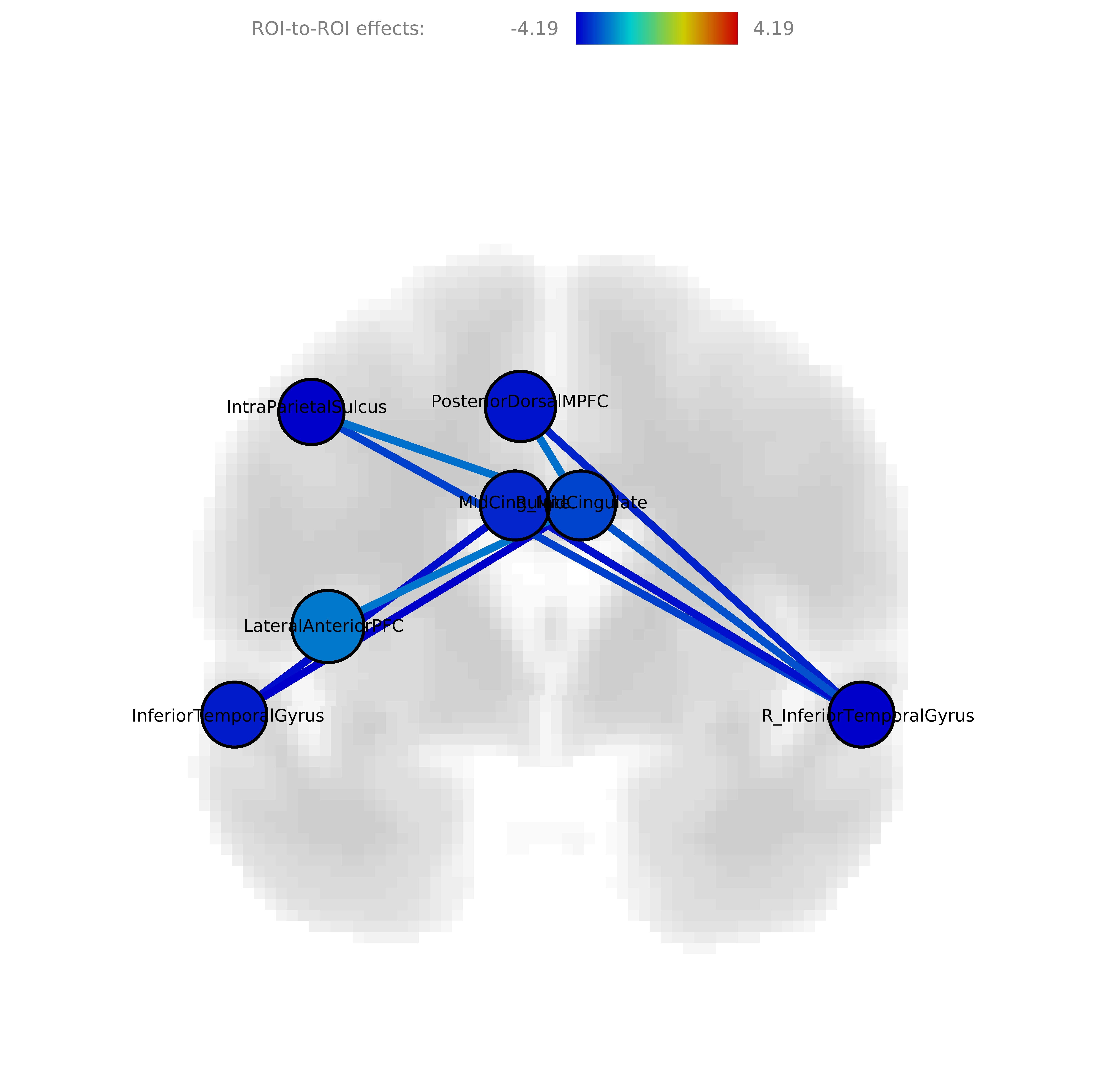

Supplement: Supplementary Figure 3 — Coronal image displaying functional connections between ROIs within the FPCN that were significantly different in terms of strength between young and older adults and passed correction for false discovery rate; blue connections indicate stronger functional connectivity among young adults. [file Image_3.jpg]

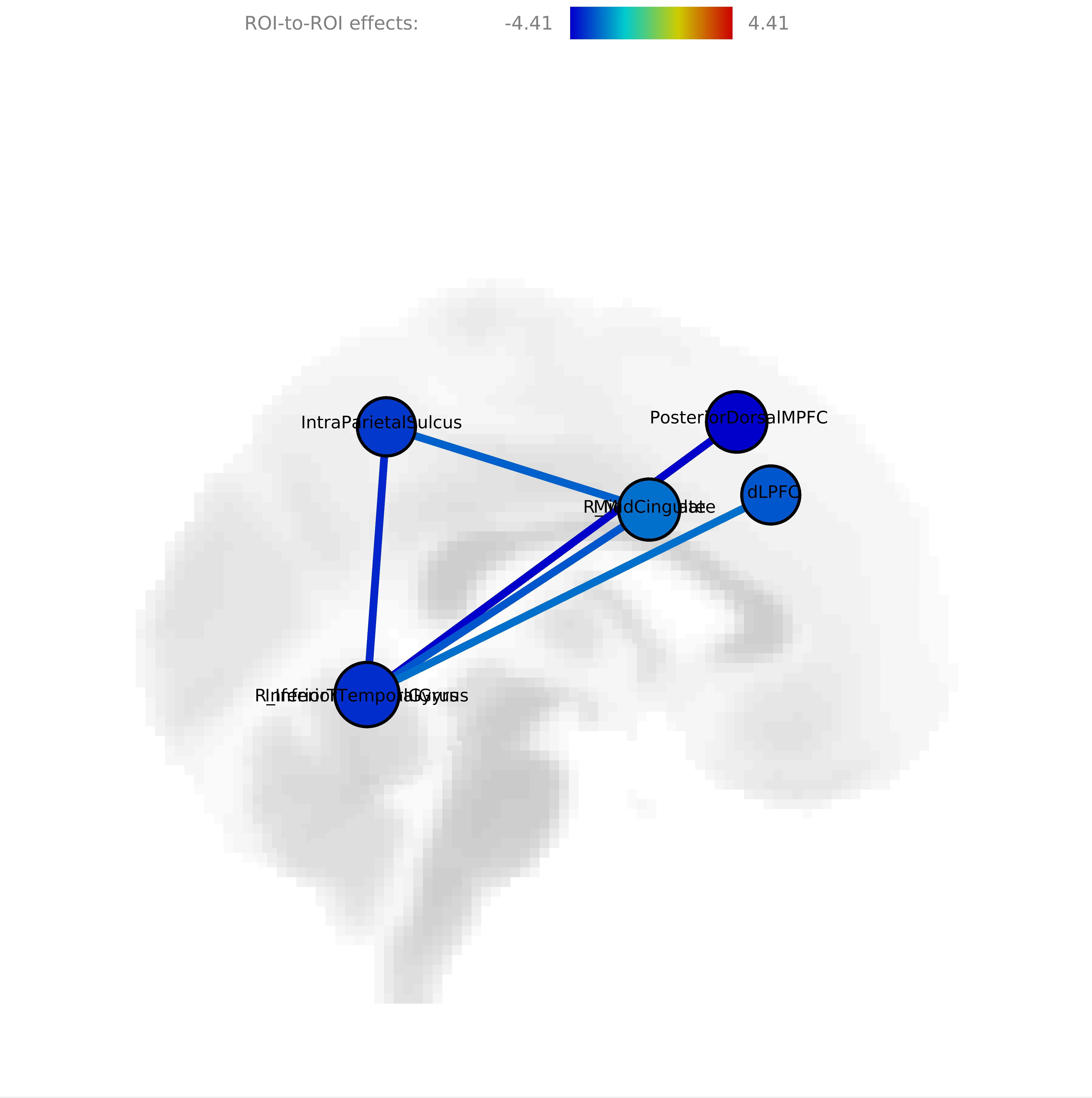

Supplement: Supplementary Figure 4 — Sagittal image displaying functional connections between ROIs within the FPCN that were significantly different in terms of strength between young and older adults when controlling for VO2 peak and passed correction for false discovery rate; blue connections indicate stronger functional connectivity among young adults. [file Image_4.jpg]

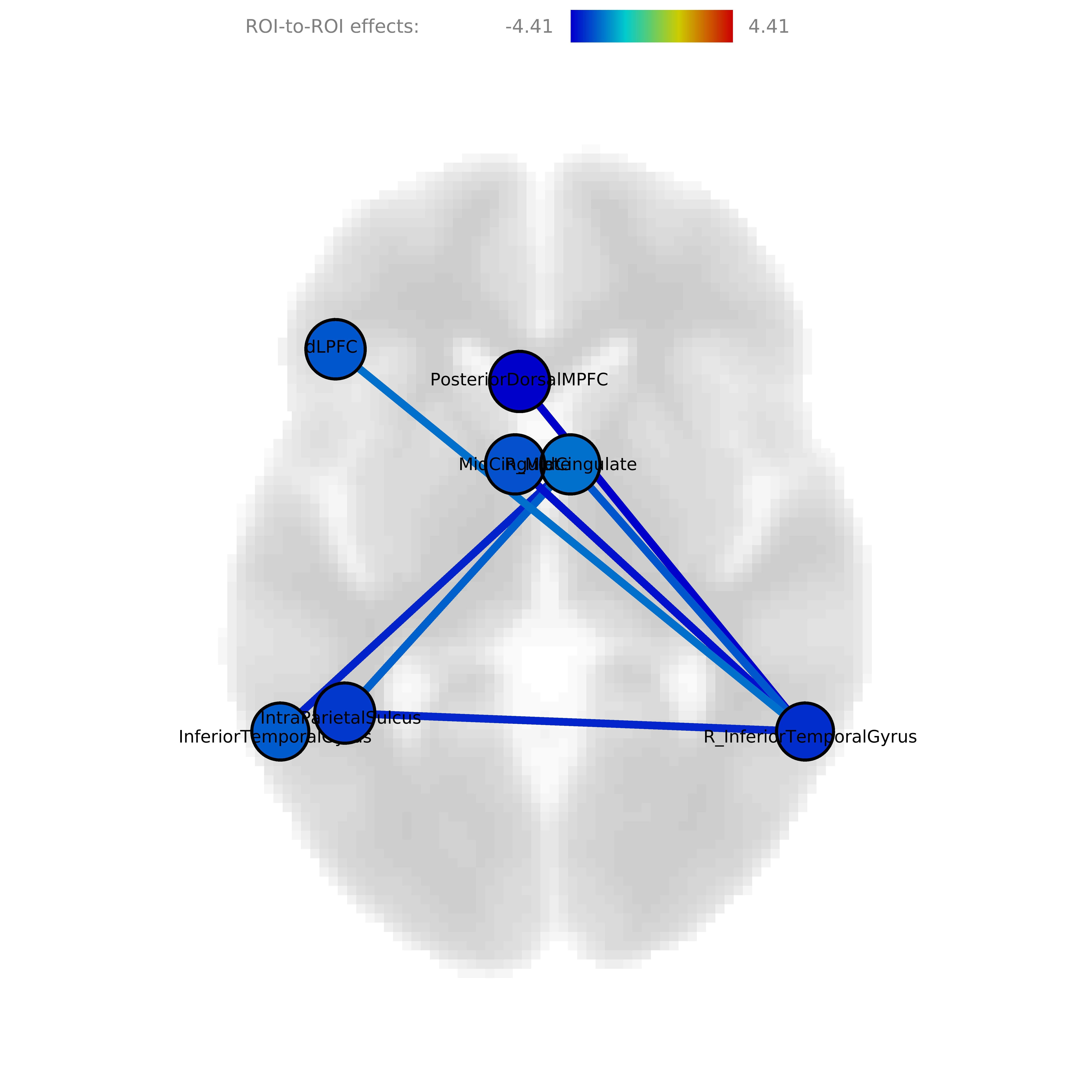

Supplement: Supplementary Figure 5 — Transverse image displaying functional connections between ROIs within the FPCN that were significantly different in terms of strength between young and older adults when controlling for VO2 peak and passed correction for false discovery rate; blue connections indicate stronger functional connectivity among young adults. [file Image_5.jpg]

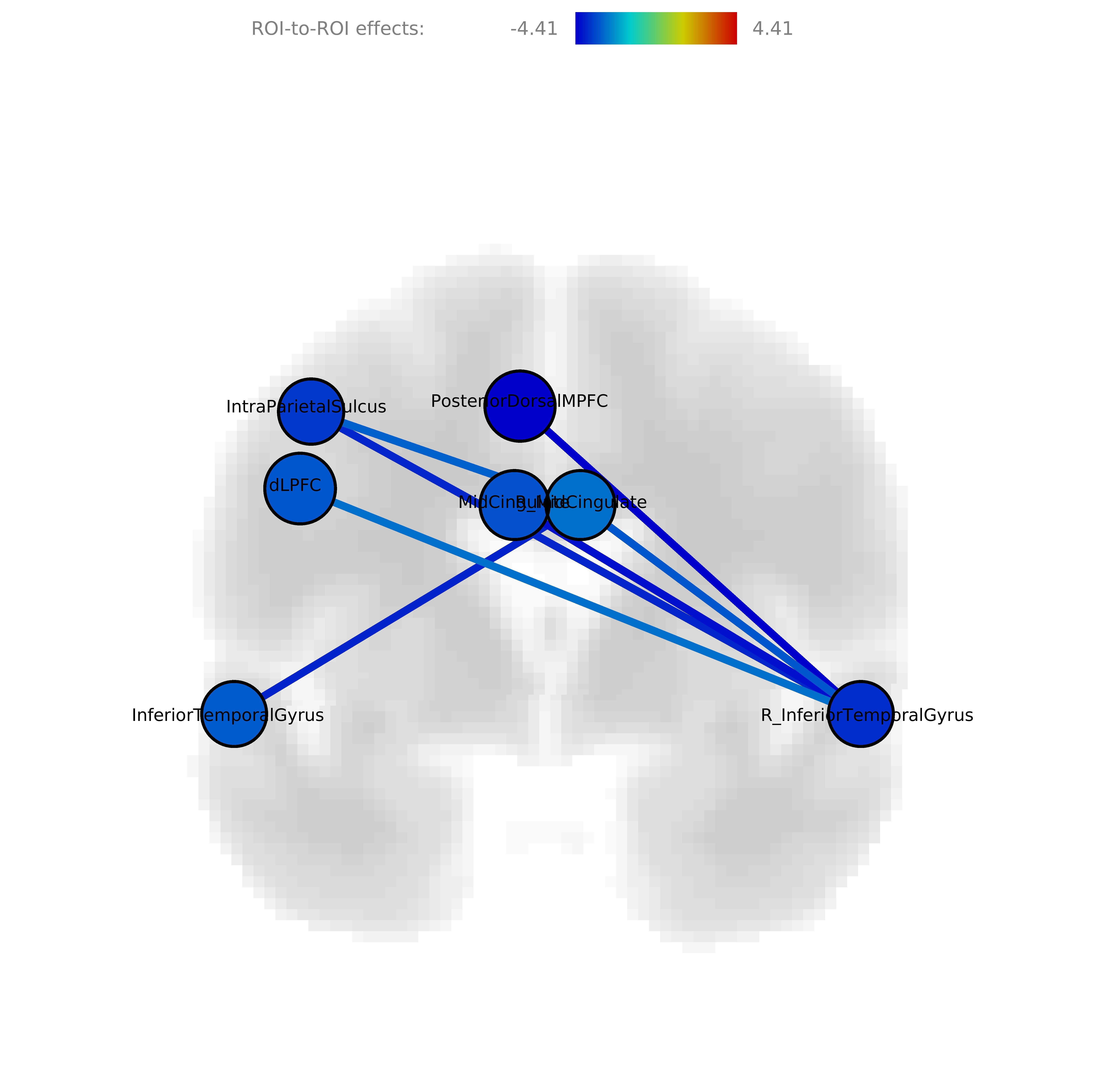

Supplement: Supplementary Figure 6 — Coronal image displaying functional connections between ROIs within the FPCN that were significantly different in terms of strength between young and older adults when controlling for VO2 peak and passed correction for false discovery rate; blue connections indicate stronger functional connectivity among young adults. [file Image_6.jpg]

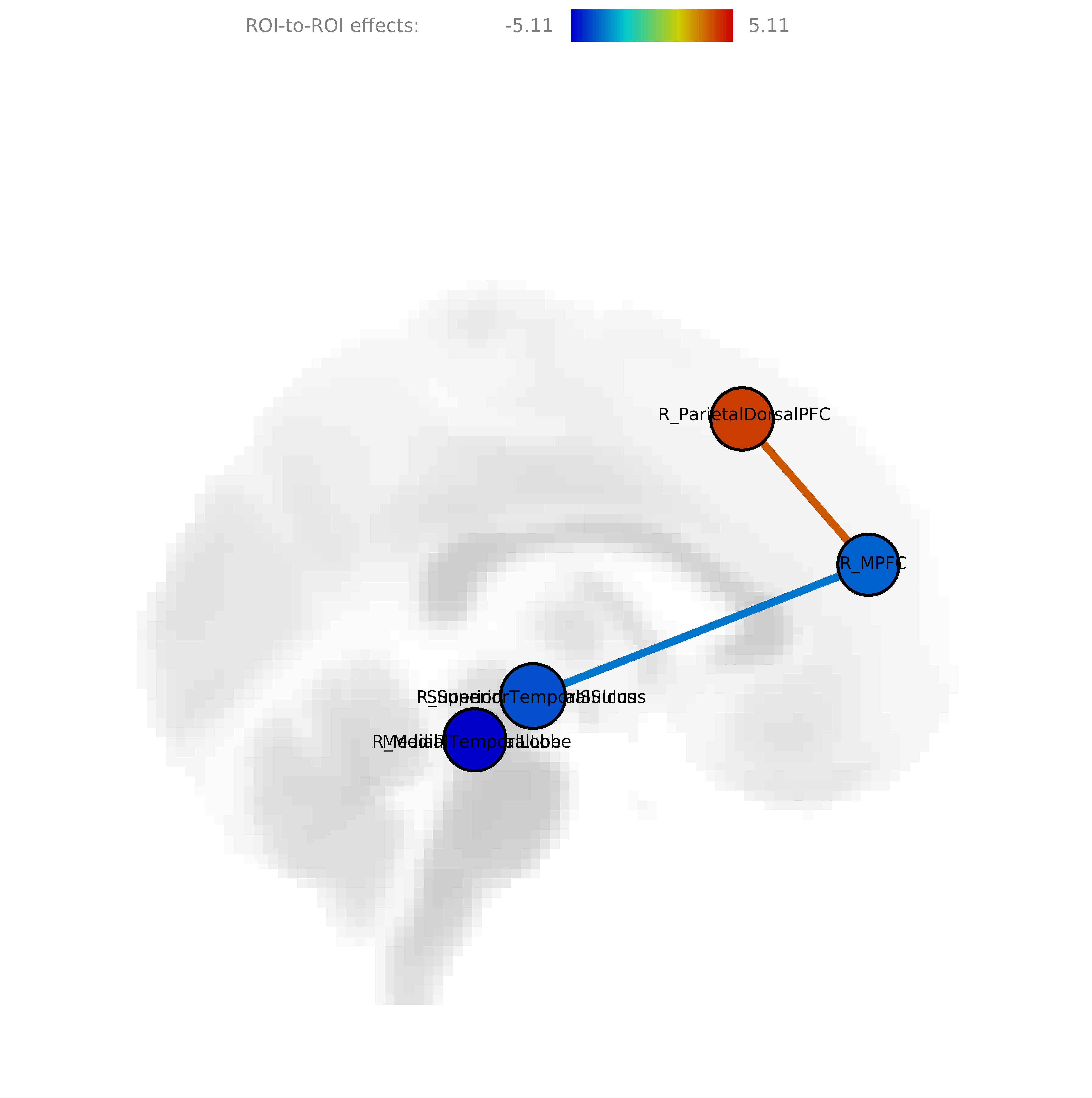

Supplement: Supplementary Figure 7 — Sagittal image displaying functional connections between ROIs within the DMN that were significantly different in terms of strength between young and older adults and passed correction for false discovery rate; blue connections indicate stronger functional connectivity among young adults, while red connections indicate stronger functional connectivity among older adults. [file Image_7.jpg]

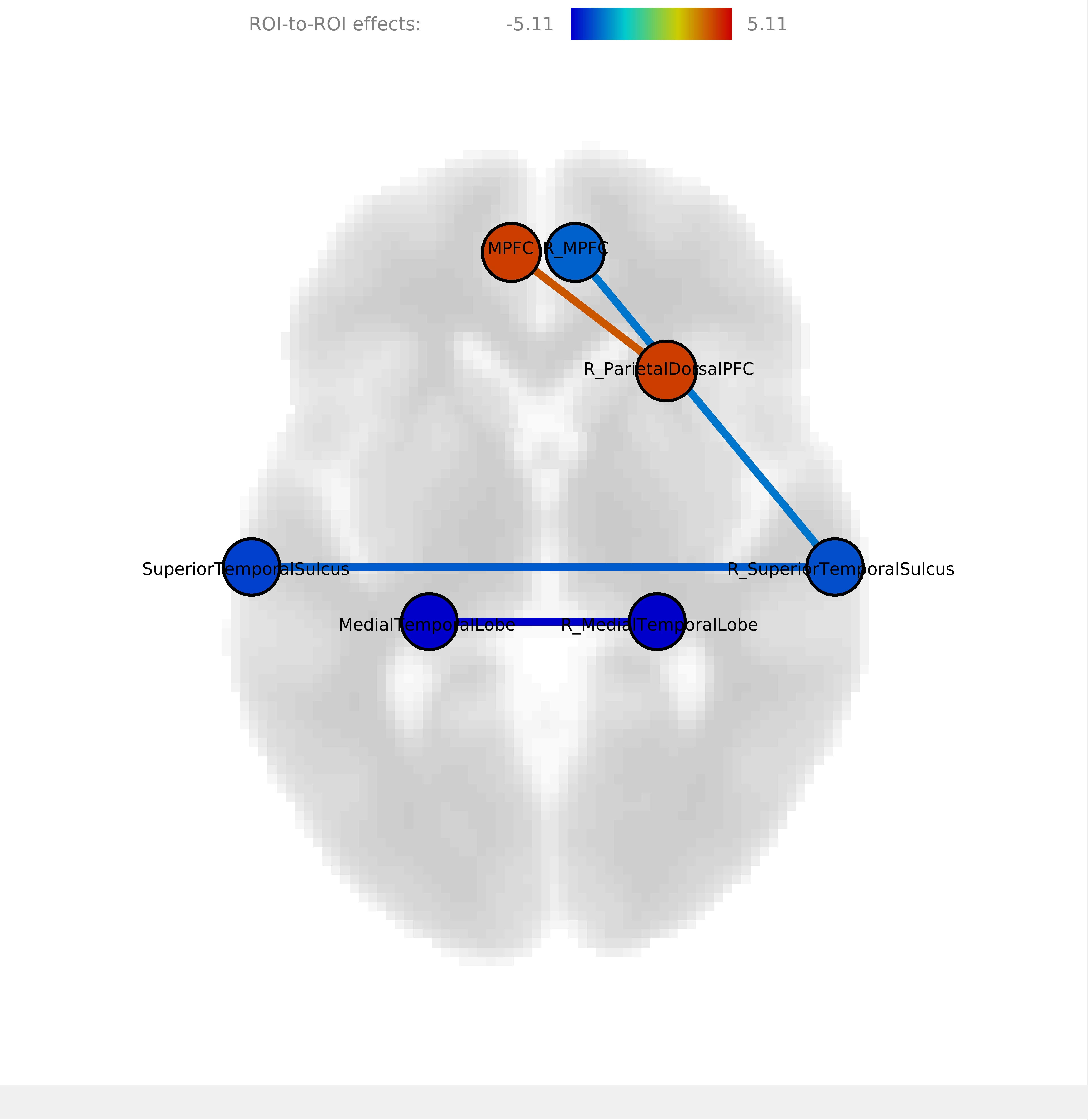

Supplement: Supplementary Figure 8 — Transverse image displaying functional connections between ROIs within the DMN that were significantly different in terms of strength between young and older adults and passed correction for false discovery rate; blue connections indicate stronger functional connectivity among young adults, while red connections indicate stronger functional connectivity among older adults. [file Image_8.jpg]

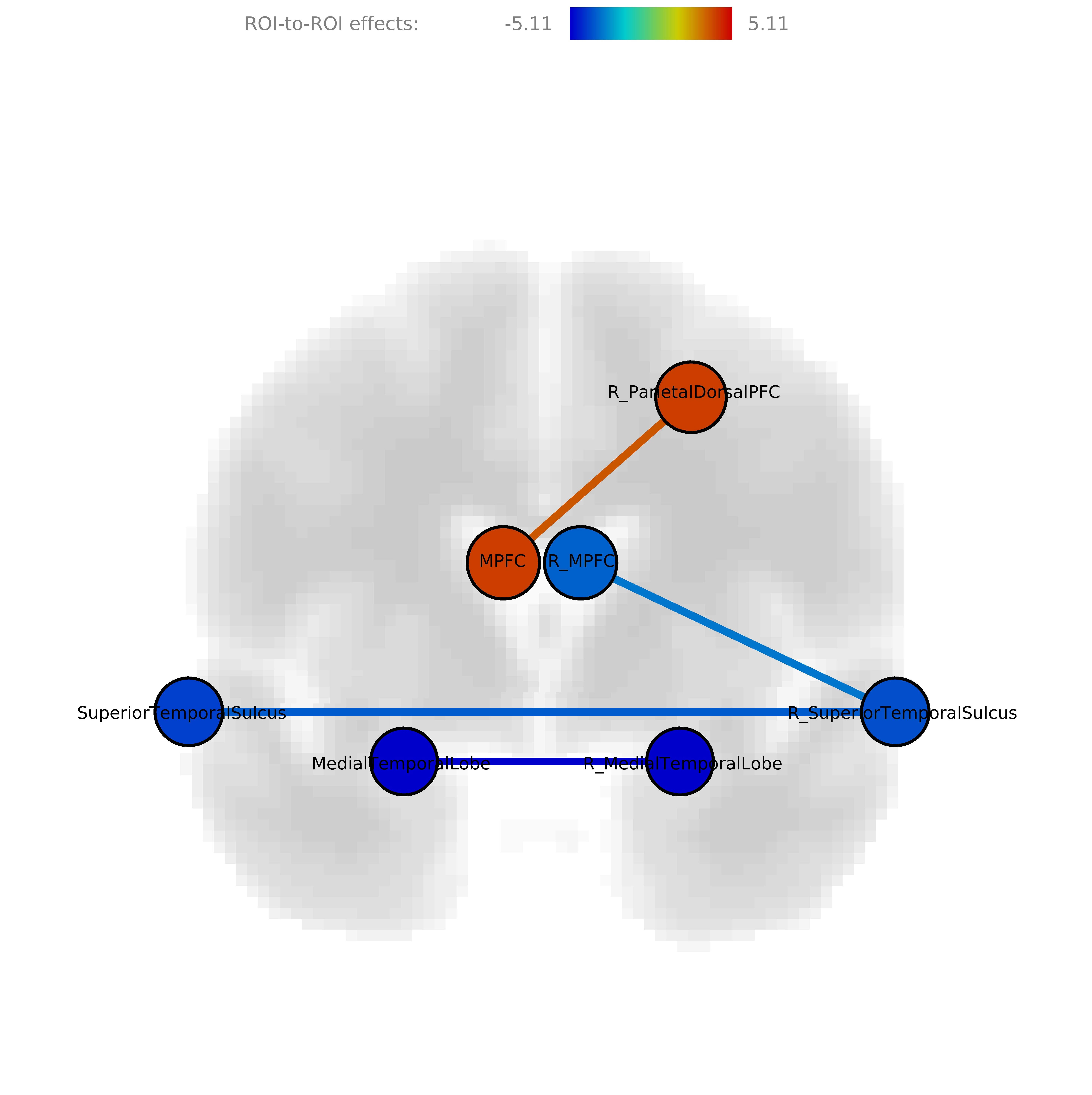

Supplement: Supplementary Figure 9 — Coronal image displaying functional connections between ROIs within the DMN that were significantly different in terms of strength between young and older adults and passed correction for false discovery rate; blue connections indicate stronger functional connectivity among young adults, while red connections indicate stronger functional connectivity among older adults. [file Image_9.jpg]

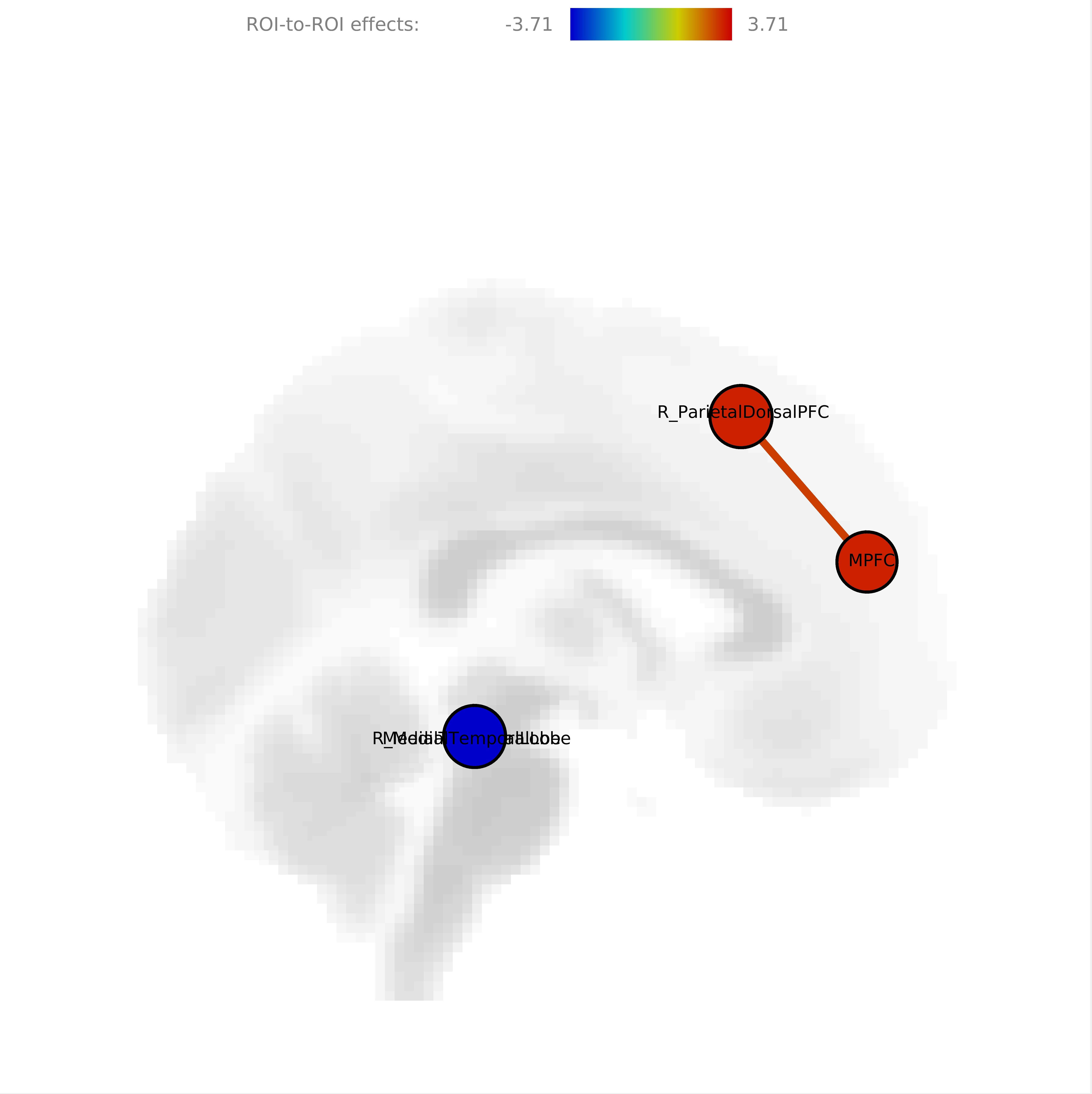

Supplement: Supplementary Figure 10 — Sagittal image displaying functional connections between ROIs within the DMN that were significantly different in terms of strength between young and older adults when controlling for VO2 peak and passed correction for false discovery rate; blue connections indicate stronger functional connectivity among young adults, while red connections indicate stronger functional connectivity among older adults. [file Image_10.jpg]

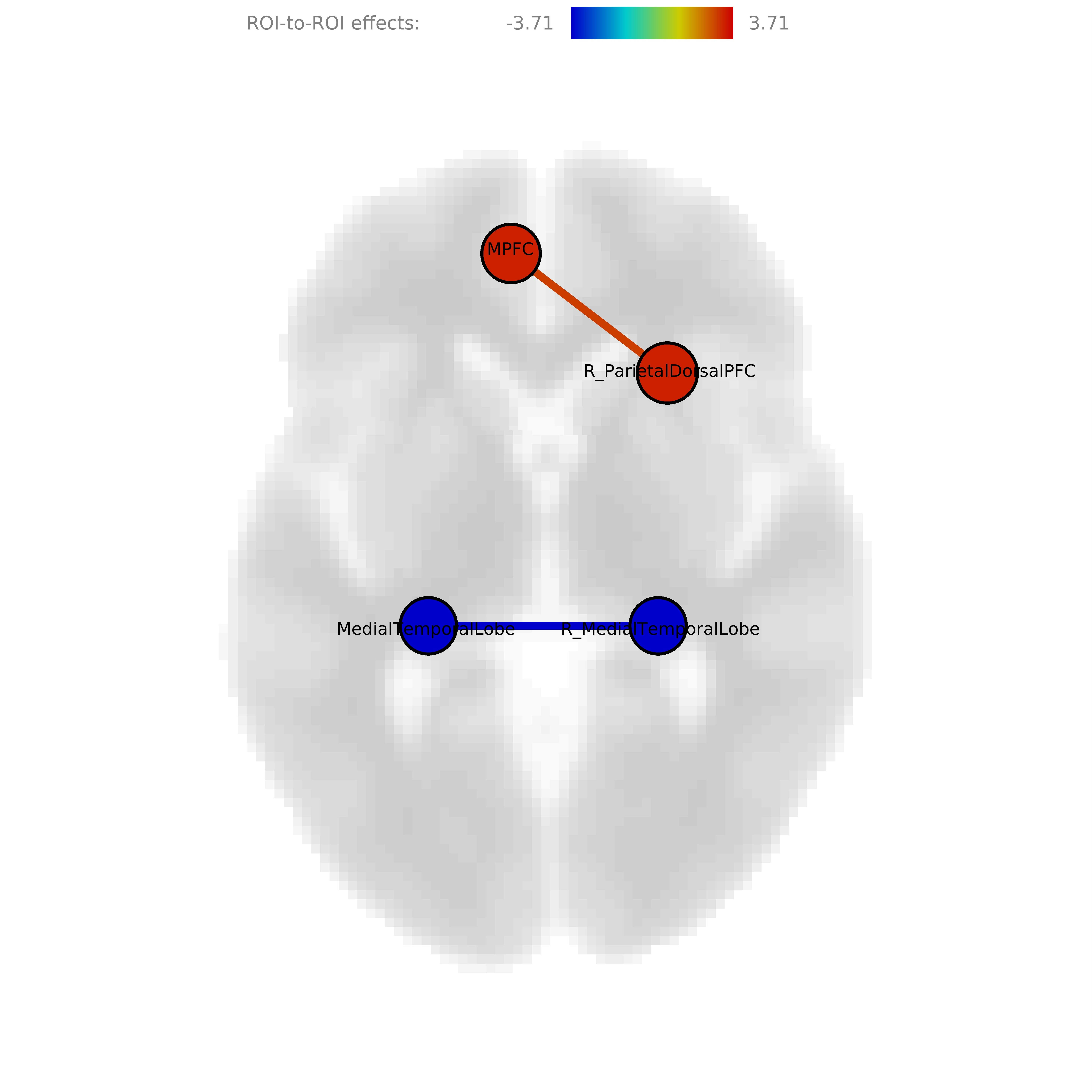

Supplement: Supplementary Figure 11 — Transverse image displaying functional connections between ROIs within the DMN that were significantly different in terms of strength between young and older adults when controlling for VO2 peak and passed correction for false discovery rate; blue connections indicate stronger functional connectivity among young adults, while red connections indicate stronger functional connectivity among older adults. [file Image_11.jpg]

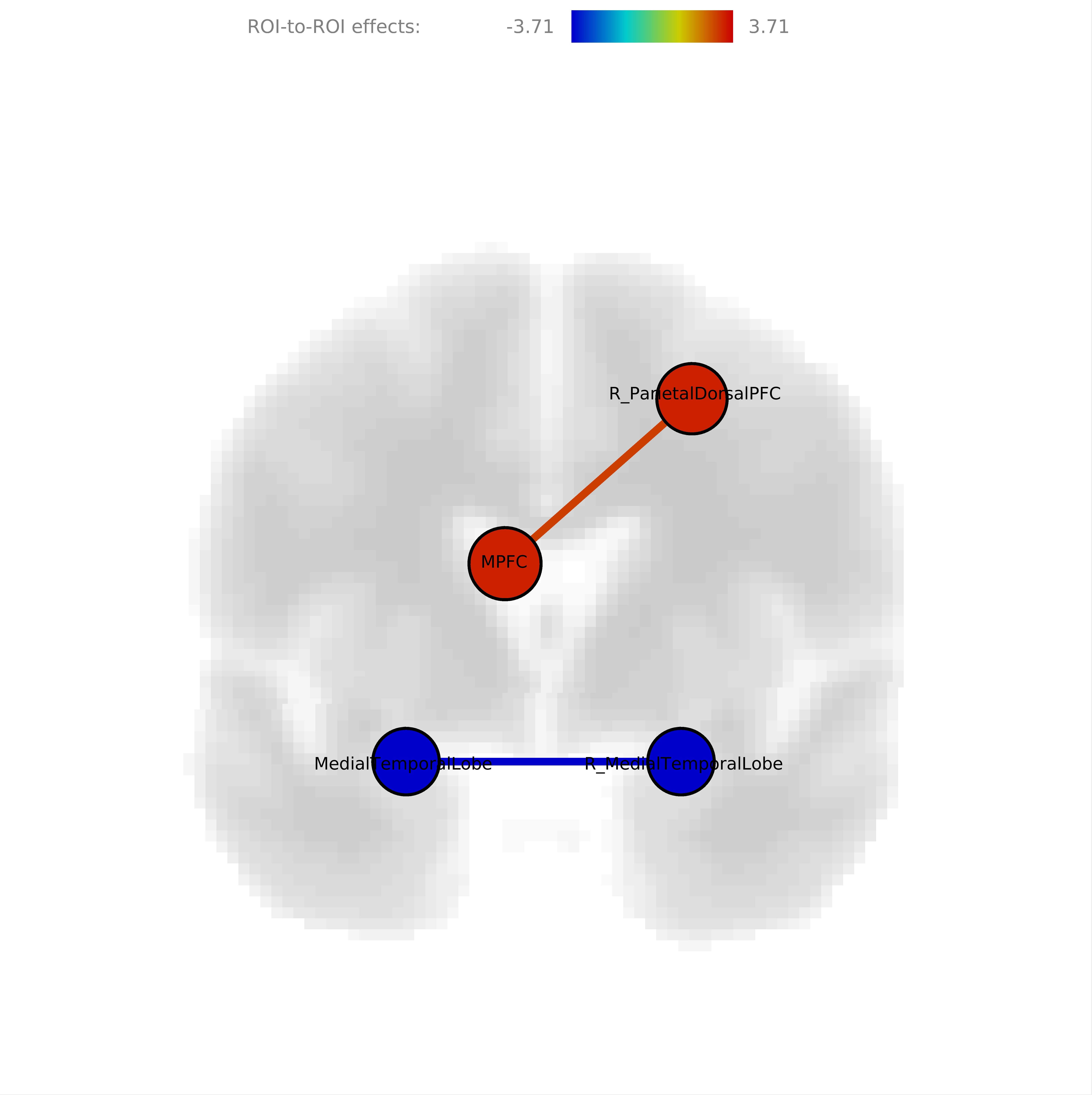

Supplement: Supplementary Figure 12 — Coronal image displaying functional connections between ROIs within the DMN that were significantly different in terms of strength between young and older adults when controlling for VO2 peak and passed correction for false discovery rate; blue connections indicate stronger functional connectivity among young adults, while red connections indicate stronger functional connectivity among older adults. [file Image_12.jpg]
